# The insulin receptor cellular IRES confers resistance to eIF4A inhibition

**DOI:** 10.7554/eLife.00542

**Published:** 2013-07-16

**Authors:** Calla M Olson, Marissa R Donovan, Michael J Spellberg, Michael T Marr

**Affiliations:** 1 Department of Biology and the Rosenstiel Basic Medical Sciences Research Center, Brandeis University, Waltham, United States; McGill University, Canada

**Keywords:** Foxo, IRES, Insulin receptor, PDCD4, eIF4A, IGFR, D. melanogaster, Mouse

## Abstract

Under conditions of stress, such as limited growth factor signaling, translation is inhibited by the action of 4E-BP and PDCD4. These proteins, through inhibition of eIF4E and eIF4A, respectively, impair cap-dependent translation. Under stress conditions FOXO transcription factors activate 4E-BP expression amplifying the repression. Here we show that *Drosophila* FOXO binds the PDCD4 promoter and stimulates the transcription of PDCD4 in response to stress. We have shown previously that the 5′ UTR of the *Drosophila* insulin-like receptor (dINR) supports cap-independent translation that is resistant to 4E-BP. Using hippuristanol, an eIF4A inhibitor, we find that translation of dINR UTR containing transcripts are also resistant to eIF4A inhibition. In addition, the murine insulin receptor and insulin-like growth factor receptor 5′ UTRs support cap-independent translation and have a similar resistance to hippuristanol. This resistance to inhibition of eIF4E and eIF4A indicates a conserved strategy to allow translation of growth factor receptors under stress conditions.

**DOI:** http://dx.doi.org/10.7554/eLife.00542.001

## eLife digest

Protein synthesis in eukaryotes occurs in two stages: transcription of DNA into messenger RNA (mRNA) in the nucleus, and then translation of that mRNA into a protein by ribosomes in the cytoplasm. These processes are regulated by a complex network of signaling pathways that enables cells to tailor protein synthesis to match current conditions. This involves regulating the expression of the genes that code for these proteins.

When cells experience stressful events, such as a shortage of oxygen or nutrients, they reduce the synthesis of most proteins. This response is regulated, in part, by a signaling pathway known as the insulin and insulin-like receptor pathway. In particular, stressful events inhibit a protein complex called eIF4F, which normally initiates the translation of mRNA molecules by binding to a structure on one end of the mRNA called the 5′ cap. Despite this general inhibition, the production of certain other proteins—including the insulin receptor itself—is actually increased in response to stress.

Olson et al. have carried out a series of experiments to explore how inhibition of the eIF4F protein complex influences the translation of the mRNA for the insulin receptor. The eIF4F complex is made up of three proteins, including one that binds to the 5′ cap and a helicase that unwinds the RNA. Previous work in the fruit fly *Drosophila* showed that translation of this mRNA can continue even if formation of the eIF4F complex is inhibited by targeting the cap binding protein. Olsen et al. now show that translation of this mRNA is also independent of the helicase. Instead, translation is maintained under these conditions because the insulin receptor mRNA contains a sequence called an internal ribosome entry site, which allows ribosomes to bind to the mRNA without the influence of the 5′ cap.

Olson et al. reveal the details of this regulatory pathway in *Drosophila* and show that similar mechanisms are at work in mammalian cells, suggesting this pathway represents a crucial regulatory process that has been conserved during evolution. A key question for future research is whether other genes within the insulin and insulin-receptor like signaling pathway use this same trick to evade translational inhibitors.

**DOI:** http://dx.doi.org/10.7554/eLife.00542.002

## Introduction

**Figure 1.**
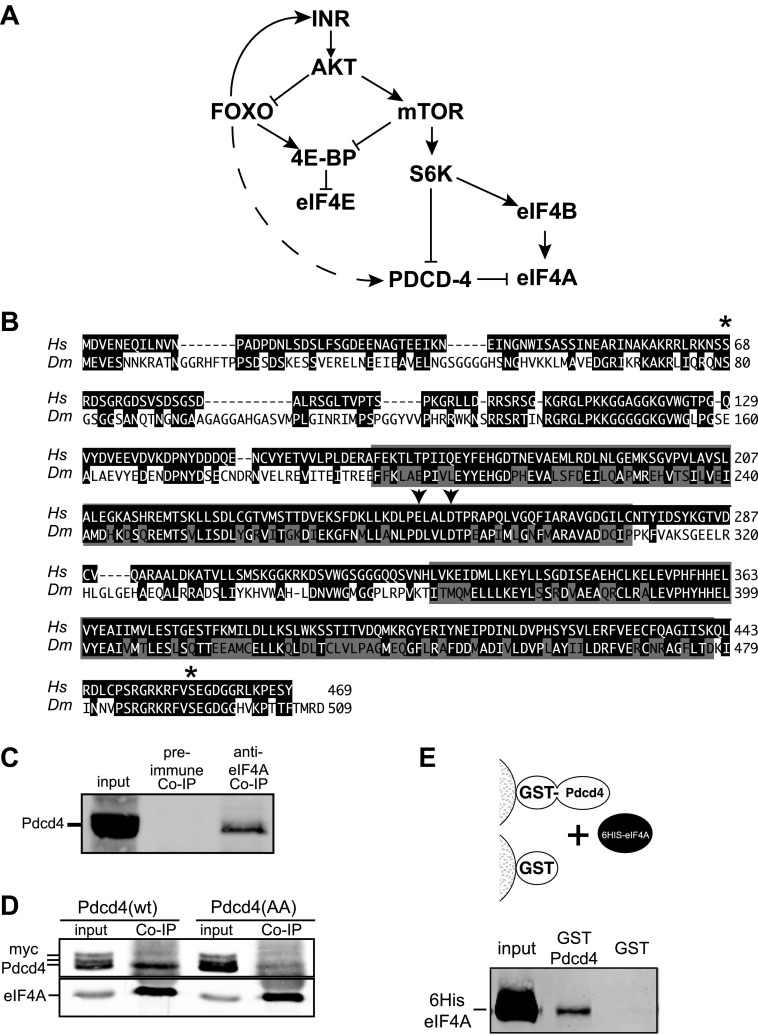
Simplified insulin/insulin-like growth factor signaling diagram. (**A**) When Insulin receptor or Insulin-like growth factor receptor is active signaling through AKT inhibits Foxo transcription factors and activates mTOR. mTOR in turn inhibits 4E-BP and activates S6K. S6K in turn inhibits Pdcd4 and activates eIF4B. When insulin signaling is low inhibition of Foxo is relieved and Foxo activates the transcription of Insulin receptor and 4E-BP. The broken line indicates the proposed activation of Pdcd4 by Foxo. (**B**) Alignment of human (*Hs* top) and *Drosophila* (*Dm* bottom) Pdcd4 proteins. Conserved Akt and S6K phosphorylation sites are indicated by asterisk. Conserved MA3 domains are indicated by shaded boxes. Arrowheads indicate conserved acidic residues important for eIF4A binding in humans. (**C**) eIF4A interacts with Pdcd4 in *Drosophila* cells. Cytoplasmic extracts from a saturated culture of S2 cells were subjected to immunoprecipitation with antisera directed against eIF4A or preimmune serum. Pdcd4 was detected with antisera against Pdcd4. (**D**) Mutant Pdcd4 binds less efficiently to eIF4A than wildtype. Cytoplasmic extracts from cultures of S2 cells expression wild-type Myc-Pdcd4 or mutant Myc-Pdcd4 (AA) were subjected to immunoprecipitation with antisera directed against eIF4A. Myc-Pdcd4 was detected with mouse monoclonal antibody to the Myc tag. Immunoprecipitated eIF4A was detected with rabbit antisera. (**E**) Immobilized *Drosophila* Pdcd4 interacts with *Drosophila* eIF4A. On the top is a cartoon of approach. On the bottom is an immnoblot of proteins eluted from the affinity columns. Position of the recombinant eIF4A is indicated. **DOI:** http://dx.doi.org/10.7554/eLife.00542.003

During times of stress the cell changes its gene expression profile to better manage the cause of the stress. Coordinate changes in both transcription and translation occur (Sengupta et al., 2010; Spriggs et al., 2010). A central pathway that responds to stress stimuli by controlling both protein and RNA synthesis is the insulin and insulin-like receptor-signaling (IIS) pathway. The fundamental molecular architecture of the IIS pathway is conserved from flies to man (Figure 1) (Oldham, 2011). When IIS signaling is high, the protein kinase AKT is activated (Ruggero and Sonenberg, 2005). AKT directly phosphorylates the Foxo family of transcription factors and consequently prevents activated transcription of Foxo target genes (Brunet et al., 1999). AKT also stimulates the activation of the mechanistic target of rapamycin (mTOR) protein (Zoncu et al., 2011).

Activated mTOR stimulates general translation, in part, by influencing the activity of the translation initiation complex eIF4F. The eIF4F complex consists of eIF4E, the 7-methyl-Guanosine-cap (m7G) binding protein, eIF4A, an RNA helicase, and eIF4G, a large scaffolding protein. In addition, the RNA binding protein eIF4B can associate with eIF4F to stimulate the helicase activity of eIF4A (Ma and Blenis, 2009; Sonenberg and Hinnebusch, 2009; Zoncu et al., 2011). mTOR stimulates general translation in part by inactivating translational inhibitors. mTOR phosphorylates and inactivates the translation repressor eIF4E binding protein (4E-BP) (Gingras et al., 1999) allowing efficient formation of the eIF4F complex. In addition, mTOR activates ribosomal protein S6 kinase (S6K) (Sarbassov et al., 2005). S6K stimulates the helicase eIF4A by activating eIF4B and inhibiting programmed cell death protein 4 (Pdcd4), a known eIF4A inhibitor (Figure 1) (Yang et al., 2003; Raught et al., 2004; Dorrello et al., 2006). Thus under conditions of high signaling through AKT and mTOR, cap-dependent translation is stimulated.

In times of stress, low levels of signaling through the IIS pathway lead to activated Foxo and 4E-BP in addition to inactive S6K. Foxo moves to the nucleus and controls the transcription of its target genes (Salih and Brunet, 2008). 4E-BP prevents formation of the translation initiation complex eIF4F, thereby inhibiting m7G-dependent translation, and S6K no longer stimulates eIF4A. This in turn leads to lower levels of global protein synthesis. Thus the IIS pathway controls gene expression with two different branches: transcription of Foxo target genes and m7G-cap-dependent translation through 4E-BP and S6K.

The IIS pathway in *Drosophila* contains a mechanism that functionally couples activated transcription to translation. A portion of the system includes a signaling and gene expression feedback loop for direct genetic targets of *Drosophila* Foxo. The insulin-like receptor (INR) and 4E-BP genes are conserved transcriptional targets of Foxo (Figure 1) (Puig et al., 2003; Puig and Tjian, 2005; Marr et al., 2007; Hu et al., 2008). Paradoxically, the insulin receptor protein, as well as mRNA, is being synthesized and accumulating under the same conditions when 4E-BP activity and expression is induced and S6K is inhibited (Marr et al., 2007).

Foxo activates the transcription of the *Drosophila* insulin receptor gene from three promoters. Each promoter produces a transcript with a distinct 5′ untranslated region (UTR) but identical coding region (Casas-Tinto et al., 2007; Marr et al., 2007). Transcripts derived from promoter 1 are by far the most abundant and ubiquitous form of INR transcript (Casas-Tinto et al., 2007; Marr et al., 2007). The *Drosophila* INR 5′ UTRs contain an internal ribosome entry site (IRES) that allows the message to escape 4E-BP inhibition of cap-dependent translation. This mechanism provides a functional coupling of transcription and translation in times of stress that allows amplification of insulin receptor expression (Marr et al., 2007). Because IRES containing transcripts can outcompete cap-dependent transcripts under these conditions their translation is actually stimulated (Svitkin et al., 2005; Marr et al., 2007). This leads to an effective switch of the cellular translation machinery to targets of the IIS pathway. Thus, Foxo targets impose a translational program by activation of genes that repress general translation while simultaneously activating targets that are immune to this translational control. This provides these targets with a competitive advantage allowing them to utilize the translation machinery that is freed by the general inhibition. Here we identify *Drosophila* Pdcd4 as an additional Foxo target further enhancing the coupling of transcription and translation regulation in the IIS pathway.

Since the IIS pathway targets translation initiation through control of both eIF4E and eIF4A, we wondered if the most abundant and ubiquitous *Drosophila* INR 5′ UTR would also provide resistance to inhibition of eIF4A activity. To answer this question we used both an in vitro translation system and a cell based assay to investigate the eIF4A requirements for efficient translation of reporters containing the INR 5′ UTR from *Drosophila*. Because mammalian systems show the same type of regulation, we also investigated the role of eIF4A inhibition in the murine insulin receptor and insulin-like growth factor receptors. (Giraud et al., 2001; Meng et al., 2008; Spriggs et al., 2009b). We find, in both the *Drosophila* and mouse systems, that the 5′ UTRs of the mRNAs for these receptors provide resistance to both eIF4E and eIF4A inhibition. Taken together, these results indicate that these cellular messages have some of the lowest requirement for eIF4F activity for translation identified to date.

## Results

### Foxo activates Pdcd4 in *Drosophila* cells

A connection between Foxo activation and translation inhibition was identified when it was discovered that 4E-BP expression is controlled by Foxo in *Drosophila* and mouse cells (Junger et al., 2003; Puig et al., 2003; Hu et al., 2008). Since the IIS pathway is also known to control eIF4A activity through Pdcd4 (Figure 1A), we tested if this gene is under direct Foxo control in *Drosophila*. Blast analysis of human Pdcd4 with the *Drosophila* genome identifies a single homologous protein encoded by CG10990. Alignment of human PDCD4 and CG10990 indicate that important regions of the protein are conserved (Figure 1B) (Cash and Andrews, 2012). The two MA3 domains, including the acidic residues shown to be important for the interaction with eIF4A are conserved (Chang et al., 2009; Waters et al., 2011). In addition, the Akt and S6K phosphorylation sites are conserved (Palamarchuk et al., 2005; Dorrello et al., 2006).

To determine if the interaction with eIF4A is conserved, we immunoprecipitated *Drosophila* eIF4A from cytoplasmic extracts derived from a saturated culture of *Drosophila* S2 cells. Associated Pdcd4 was detected by immunoblot. Pdcd4 is co-precipitated with antisera against eIF4A but not with preimmune serum indicating that Pdcd4 and eIF4A interact in *Drosophila* cells (Figure 1C). We next created Myc-tagged expression constructs for *Drosophila* Pdcd4, one wildtype construct and a construct containing mutations in conserved residues in the first MA3 domain (E282A, D286A). The analogous mutations in human PDCD4 destabilize the interaction with eIF4A (Chang et al., 2009; Waters et al., 2011). The constructs were transfected into growing *Drosophila* S2 cells. Subsequent immunoprecipitation of eIF4A from these cells reveals a decreased association of the mutant Pdcd4 with eIF4A (Figure 1D). This indicates the mutations induce the same destabilization of the eIF4A–Pdcd4 interaction in *Drosophila* cells. Interestingly under these conditions we detect multiple forms of Pdcd4 by western blot, most likely phosphorylated forms of Pdcd4, and only the fastest migrating species associates with eIF4A.

To determine if *Drosophila* Pdcd4 can interact directly with eIF4A we used an affinity chromatography assay using recombinant proteins purified from *Escherichia coli*. A GST fusion to *Drosophila* Pdcd4 was immobilized on glutathione agarose and recombinant *Drosophila* eIF4A was passed over the column (Figure 1D). As a control GST was also immobilized on glutathione agarose. The eIF4A bound to the immobilized GST-Pdcd4 but not to GST alone indicating that *Drosophila* Pdcd4 can interact directly with *Drosophila* eIF4A. Taken together these data are consistent with the notion that CG10990 is the *Drosophila* homologue of human PDCD4.

**Figure 2.**
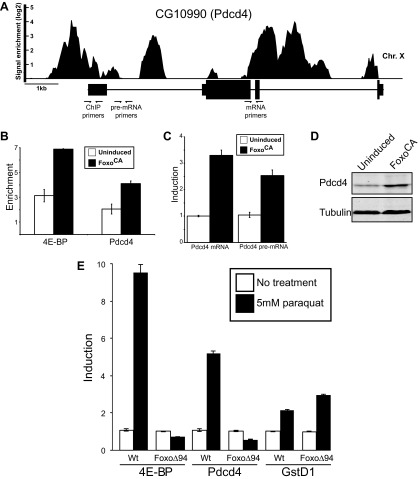
Foxo activates Pdcd4 in *Drosophila* cells. (**A**) Reanalysis of ChIP-chip data from Teleman et al. (2008). Genomic Browser view of Foxo binding to the Pdcd4 locus in starved larva. The data are plotted as the enrichment (log2) over mock precipitated samples. Primers used for ChIP and qPCR are indicated. (**B**) ChIP of Foxo at 4E-BP promoter and Pdcd4 locus in *Drosophila* S2 cells expressing constitutively active Foxo (Foxo^CA^). The data are plotted as fold enrichment over a background region 1 kb downstream of 4E-BP. Uninduced samples are plotted in white, induced samples in black (error bars indicate SD). (**C**) RT-qPCR of Pdcd4 mRNA and pre-mRNA in *Drosophila* S2 cells expressing Foxo^CA^. Data are plotted as fold-induction (error bars indicate SD). (**D**) Immunoblot of total protein from *Drosophila* S2 cells expressing Foxo^CA^. Positions of Pdcd4 and tubulin are indicated. (**E**) 4E-BP, GstD1, and Pdcd4 RNA levels in untreated and paraquat-treated animals. The levels of RNA were normalized to RP49 and are plotted as fold-induction relative to untreated animals (error bars indicate SEM). **DOI:** http://dx.doi.org/10.7554/eLife.00542.004

There are hints in the literature, based on microarray experiments, indicating this gene is induced in response to nutrient stress and might be controlled by Foxo (Gershman et al., 2007). In an effort to determine if Foxo binds to the Pdcd4 gene in nutrient stressed animals we reanalyzed the only publically available Foxo ChIP (Chromatin immunoprecipitation) dataset (Teleman et al., 2008). These experiments were performed on starved larva. We find Foxo binds the Pdcd4 gene in both the promoter and intronic regions with enrichment values as high as 16-fold over background (Figure 2A). To corroborate this finding, we performed ChIP on genomic DNA from a cell line with an inducible Foxo cDNA gene that has been modified so the Foxo protein produced is constitutively active because it is immune to the negative regulation by insulin signaling (Foxo^CA^) (Puig et al., 2003; Gershman et al., 2007). This allows us to induce Foxo under conditions of high nutrient signaling and remove possible crosstalk from upstream signaling pathways. We tested the enrichment of genomic sequences by qPCR using primers to the Pdcd4 promoter region (Figure 2A) compared to a region in the first intron of CG15414, a gene just downstream of 4E-BP. We find Foxo^CA^ binds to the promoter region of Pdcd4 at levels comparable to a well-defined direct target, 4E-BP (Junger et al., 2003; Puig et al., 2003; Marr et al., 2007) (Figure 2B). To determine the effect on mRNA production under these conditions we performed quantitative RT-qPCR on induced cells. We find that the steady-state level of Pdcd4 mRNA is increased about threefold in cells expressing active Foxo (Figure 2C). To determine if the effect is due to mRNA stability changes or new transcription we assayed intron-containing pre-mRNAs by RT-qPCR. Since most splicing is co-transcriptional in *Drosophila* this is a good assay for new RNA synthesis (Khodor et al., 2011). We find that Pdcd4 pre-mRNA is increased, indicating an increase in transcription of the gene. The increased mRNA also leads to increased protein synthesis as determined by immuno-blot with antibodies directed against *Drosophila* Pdcd4 (Figure 2D). This is likely an underestimate of the effect since these experiments are all done under high serum and insulin conditions that should result in the rapid turnover of Pdcd4 protein (Dorrello et al., 2006).

We tested if Foxo controls Pdcd4 in adult flies subjected to stress. Adult wildtype or Foxo null flies (Slack et al., 2011) were treated with paraquat to induce oxidative stress and assayed for expression of 4E-BP, Pdcd4, and GstD1 by RT-qPCR. Consistent with previous results (Wang et al., 2005), 4E-BP is induced by paraquat in a Foxo dependent manner. Like 4E-BP, we find that Pdcd4 is induced in response to paraquat in a Foxo dependent manner (Figure 2E). To determine if the effect was due to loss of general oxidative stress response or if it is Foxo specific we examined the induction of GstD1, a gene controlled by Nrf2 in response to oxidative stress (Misra et al., 2011). We find that GstD1 is still responsive indicating that the effects at Pdcd4 and 4E-BP are Foxo-specific and not due to a loss of responsiveness in the mutant flies (Figure 2E). These results are consistent with the idea that in addition to controlling the cap-binding complex through 4E-BP, active Foxo can influence eIF4A through activation of the Pdcd4 gene in response to stress. Given that Foxo is activating transcription of the INR gene while Pdcd4 is also active, we hypothesized that since the INR mRNA is translated efficiently under these conditions (Marr et al., 2007) it must be at least partially immune to diminished eIF4A activity.

### Translation dependent on the *Drosophila* insulin receptor 5′ UTR is stimulated by Pdcd4

**Figure 3.**
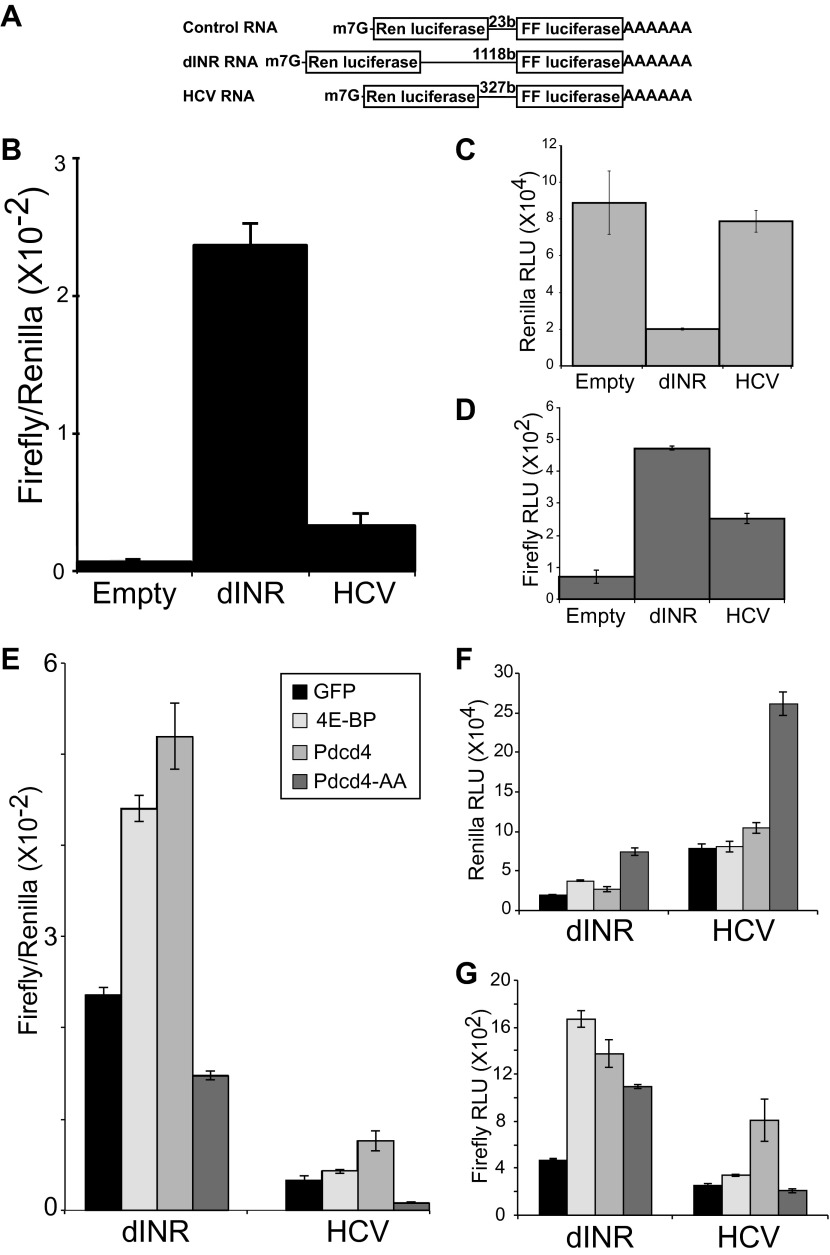
*Drosophila* insulin receptor 5′UTR provides resistance to Pdcd4. (**A**) Diagram of dicistronic reporters. Translation of the Firefly ORF requires internal ribosome entry. Firefly to renilla activity ratio provides an indication of IRES activity. (**B**) The *Drosophila* Insulin receptor UTR provides IRES activity comparable to the activity of HCV. (**C**) Renilla activity of the reporters. (**D**) Firefly activity of the reporters. (**E**) Dicistronic reporter activities in the presence of 4E-BP or Pdcd4 expression. 4E-BP and Pdcd4 stimulate the IRES activity of the insulin receptor UTRs. Mutation of the critical acidic residues of Pdcd4 prevents the stimulation. (**F**) Renilla activity of the reporters in the presence of expressed proteins. (**G**) Firefly activity of the reporters in the presence of the expressed proteins (error bars indicate SEM). **DOI:** http://dx.doi.org/10.7554/eLife.00542.005

To determine the effect of increased Pdcd4 on the translation of insulin receptor UTR containing RNAs we modified a dicistronic mRNA assay which we previously used to investigate the effects of 4E-BP on insulin receptor translation in *Drosophila* (Marr et al., 2007). In this assay a construct is used that produces a RNA in which the open reading frames of renilla luciferase and firefly luciferase are present on the same transcript (Figure 3A). Renilla luciferase levels are an indication of total message produced in the cell and firefly luciferase levels are an indication of IRES dependent translation. As previously reported, insertion of the INR 5′ UTR between the ORFs promotes translation of the second ORF (Marr et al., 2007). The levels of IRES activity of the INR UTR are comparable to the well-characterized IRES from Hepatitis C Virus (Figure 3B) (Tsukiyama-Kohara et al., 1992). The INR UTR and the HCV IRES both produce more firefly signal than the empty vector (Figure 3D).

To determine the effect of Pdcd4 on the INR UTR we expressed Pdcd4 in *Drosophila* S2 cells and measured the activity of the dicistronic reporter. In order to more accurately determine the effects of the protein in question we modified the assay so the production of the dicistronic mRNA is inducible. This allows accumulation of the experimental protein in the cell before the dicistronic mRNA is produced giving a more precise measure of the effects on the activity of the dicistronic message. The levels of expression of the second ORF relative to the first ORF are an indication of the cap-independent translation potential of the insert. To validate this assay we reproduced our previous results with 4E-BP (Marr et al., 2007). As reported previously, expression of 4E-BP stimulates the translation of the second open reading frame in a reporter containing the INR 5′UTR (Figure 3E,G). Expression of Pdcd4 also stimulates translation of the second ORF dependent on the INR 5′UTR similar to the effects seen with the HCV IRES (Figure 3E,G). These effects are specific. Mutation of the key acidic residues (Figure 1B) in Pdcd4 shown to disrupt eIF4A binding in the human system (Waters et al., 2011) prevent the IRES stimulation.

### Drosophila insulin receptor 5′ UTR1 is resistant to eIF4A inhibition

To address the role of eIF4A in insulin receptor translation, we used a highly specific small molecule inhibitor of eIF4A, hippuristanol (Bordeleau et al., 2006; Lindqvist et al., 2008). Hippuristanol is a potent translation inhibitor that works in eukaryotes from yeast to human (Lindqvist et al., 2008). Hippuristanol inhibits the ATPase activity and RNA binding of eIF4A (Bordeleau et al., 2006; Lindqvist et al., 2008). The small molecule binds to the protein in conserved regions V and VI in eIF4A homologues (Lindqvist et al., 2008). Importantly, the effects on translation can be rescued by addition of either wild-type or mutant forms of eIF4A that are immune to hippuristanol indicating that the effects are highly specific for eIF4A (Bordeleau et al., 2006; Lindqvist et al., 2008). This small molecule had been used previously in the *Drosophila* system to investigate eIF4A requirements (Iwasaki et al., 2009).

**Figure 4.**
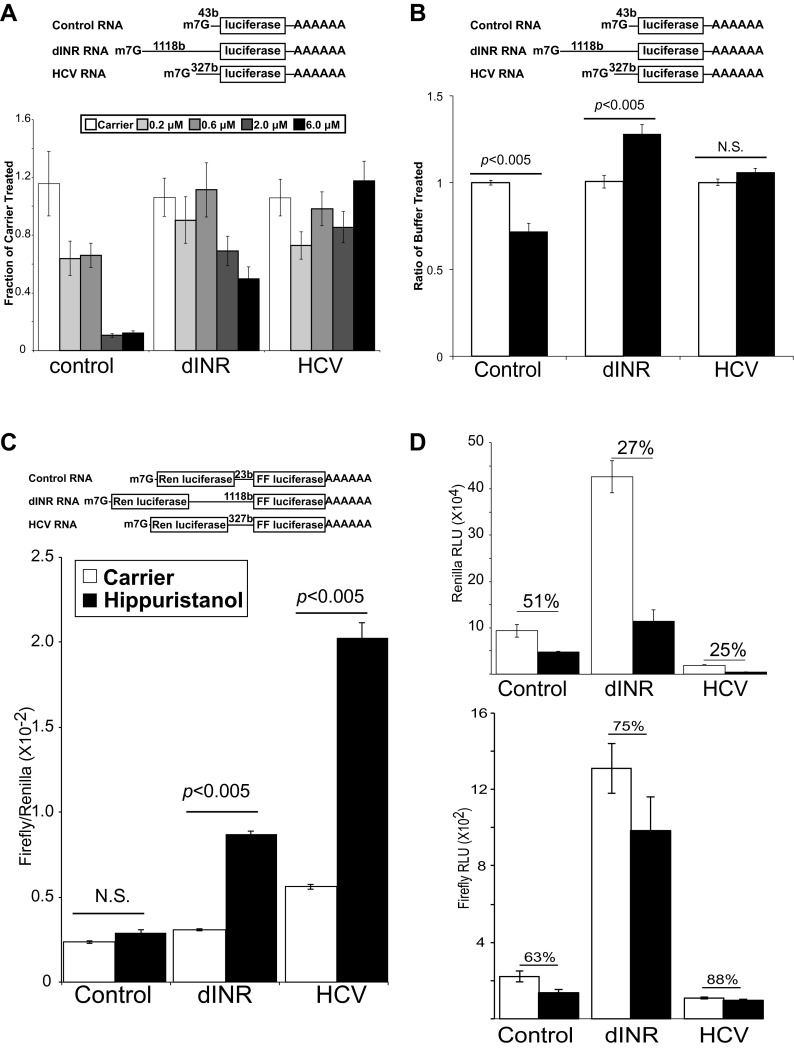
*Drosophila* insulin receptor 5′UTR provides resistance to eIF4a inhibition. (**A**) Top: Diagram of RNAs used in the in vitro translation assays. Bottom: Titration of hippuristanol in in vitro translation assays. The shade of the bars indicates the final concentration of hippuristanol in the assay. The legend appears above the graph. Data are plotted as the fraction activity of the carrier treated extracts (error bars indicate SEM). (**B**) Top: Diagram of RNAs used in the in vitro translation assays. Bottom: Activity of these RNAs in in vitro translation assays in the absence (white bars) and presence (black bars) of *Drosophila* Pdcd4 (error bars indicate SEM). (**C**) Dicistronic RNA translation in vitro. Top: Diagram of RNAs used in the in vitro translation assays. Bottom: Firefly to renilla ratio in the absence (white bars) and presence (black bars) of hippuristanol. (**D**) Top: Renilla activity in the dicistronic assay. Bottom: Firefly activity in the dicistronic assay. Shading as in **C**. Percentage above the bars indicates activity after hippuristanol addition relative to carrier treated samples. **DOI:** http://dx.doi.org/10.7554/eLife.00542.006

We performed in vitro competitive translation experiments with capped and polyadenylated firefly luciferase reporters (Figure 4A) and a *Drosophila* embryo extract translation system that has not been treated with micrococcal nuclease (Gebauer et al., 1999; Marr et al., 2007) in the presence of hippuristanol. The RNA reporters contain the 5′ UTR from the *Drosophila* insulin receptor. In addition we include two control RNAs. One control RNA contains a non-specific UTR derived from plasmid sequences. The other RNA contains the IRES from the Hepatitis C virus (HCV) (Tsukiyama-Kohara et al., 1992). This IRES does not require eIF4A activity and controls for non-specific effects on the extract (Pestova et al., 1998). Under the experimental conditions, translation of the first control RNA is strongly inhibited by hippuristanol while the reporter containing the HCV IRES is completely resistant to eIF4A inhibition (Figure 4A). This small molecule inhibitor exposed a greatly diminished role for eIF4A in the *Drosophila* INR UTR mediated translation (Figure 4A). At the low and moderate concentrations of hippuristanol, the *Drosophila* INR UTR reporter retains almost complete activity comparable to the HCV UTR. Even at the highest concentration of hippuristanol tested, the reporter containing the INR UTR retains >50% of the original translation activity. To determine the effect of Pdcd4 in this system we added recombinant Pdcd4 to the translation extract. Consistent with the data using hippuristanol, we find Pdcd4 can inhibit the control RNA but not the *Drosophila* INR UTR or the HCV IRES (Figure 4B). These finding suggests that translation of the most abundant *Drosophila* INR transcript can tolerate inhibition of eIF4A.

We used a dicistronic RNA in the in vitro translation assay to directly test the IRES activity under hippuristanol treatment (Figure 4C top). Dicistronic RNAs were synthesized in vitro using T7 RNA polymerase. The RNA was capped and polyadenylated and used to program the same *Drosophila* embryo translation system described above. The extracts were treated with either hippuristanol or carrier. Consistent with the monocistronic assay, both the INR and the HCV IRES containing transcripts show increased relative translation of the second ORF upon eIF4A inhibition (Figure 4C). The increase is due both to a resistance of the second ORF to the inhibition and a decrease in the cap-dependent translation of the first ORF (Figure 4D). Under these conditions there is a small amount of cryptic translation of the second ORF in the control transcript. However both the renilla and firefly activites respond the same to the hippuristanol treatment.

### The cap-independence and eIF4A requirements of insulin receptor mRNA are conserved in mammals

**Figure 5.**
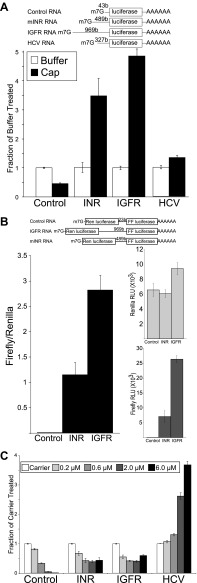
Mammalian insulin receptor and insulin-like growth factor receptor 5′UTR provide resistance to eIF4a inhibition. (**A**) Diagram of RNAs used in the in vitro translation assays. (**B**) In vitro Translation in the absence (white bars) and presence (black bars) of excess m7G analogue. Data are plotted as the fraction activity of the mock treated extracts (error bars indicate SEM). (**C**) Titration of hippuristanol in in vitro translation assays. Data are plotted as the fraction activity of the mock treated extracts (error bars indicate SEM). **DOI:** http://dx.doi.org/10.7554/eLife.00542.007

Because the molecular architecture of the IIS signaling pathway is conserved in mammals, we wondered if this level of regulation would be conserved in mammals. To address this, we cloned the 5′ UTR from the longest mRNAs for the mouse insulin receptor (mINR) and the mouse insulin-like growth factor receptor-I (IGFR) and created firefly luciferase reporters under the control of these UTRs (Figure 5A). Previously, it was reported that INR and IGFR UTRs confer cap-independent translation activity in human and rat (Giraud et al., 2001; Spriggs et al., 2009b). To extend this finding to the mouse system and ensure that our competitive rabbit reticulocyte system was capable of supporting cap-independent translation, we assayed translation of the mINR and IGFR reporters in the presence of excess m7G cap along with our control RNA and the HCV IRES reporters described above (Figure 5A). While the control RNA is inhibited by excess cap, translation from the 5′ UTR of mINR and IGFR is not only resistant to excess m7G cap but the activity actually increases in the presence of excess m7G cap. This observation is common with UTRs that contain an IRES (Svitkin et al., 2005). The increase in activity for both the mINR and IGFR UTRs exceeded the IRES activity of the HCV UTR. This indicates that mINR UTR and IGFR UTR are capable of conferring cap-independent translation initiation.

To directly test the ability of these UTRs to allow internal ribosome entry we performed a dicistronic assay in mammalian cells. The UTRs were subcloned into a plasmid construct between the renilla and firefly open reading frames controlled by the RSV LTR. Both the mINR and the IGFR UTRs supported substantial firefly activity compared to the original vector. This is apparent both in the firefly to renilla ratio and in the raw firefly activity units (Figure 5B). This combined with the in vitro translation assays strongly suggest that the mINR and IGFR UTRs can support cap-independent translation.

To test for conservation of resistance to eIF4A inhibition, hippuristanol was titrated into the rabbit reticulocyte translation system. As expected, the activity of the control RNA is reduced to <10% of the mock treated extract, and the HCV IRES is completely resistant to hippuristanol (Figure 5C). In fact, the HCV IRES is stimulated fourfold by addition of hippuristanol under these conditions. Both mINR and IGFR UTRs confer resistance to hippuristanol (Figure 5C). Even at the highest concentrations of the small molecule the mINR and mIGFR UTRs remain roughly 50% active indicating a decreased requirement for eIF4A relative to the control RNA.

## Discussion

Stress responses controlled through the IIS pathway result in changes in both mRNA synthesis and protein synthesis. These processes are coordinated to ensure proper expression of the downstream targets. Under the same low IIS signaling conditions that activate 4E-BP, Pdcd4 is stabilized resulting in the inhibition of the DEAD box helicase eIF4A. Thus, under these conditions the eIF4F complex is repressed by two mechanisms. At the same time, the Foxo family of transcription factors is active and increasing the synthesis of certain mRNAs, one of which is the INR transcript itself.

Previously we identified a connection between 4E-BP mediated inhibition of the cap-binding complex and INR mRNA translation in *Drosophila* (Marr et al., 2007). The INR message is immune to the 4E-BP translational repression and thus is preferentially translated under low signaling conditions coupling the increase in mRNA expression to an increase in protein synthesis.

In the work presented here we extend this observation of gene expression coordination of INR mRNA to the eIF4A branch of the IIS signaling pathway. First, we show that active Foxo is capable of directly stimulating the transcription of Pdcd4, analogous to the activation of 4E-BP seen previously under these same conditions (Junger et al., 2003; Puig et al., 2003). This provides a mechanism for the pathways controlling Foxo to enhance the inhibition of eIF4A when stressed or when nutrients are low. Second we show that the 5′ UTR of the most abundant *Drosophila* INR transcript provides resistance to eIF4A inhibition comparable to the resistance seen with the HCV IRES that does not require eIF4A. A similar finding has been seen for the *Drosophila* reaper 5′ UTR (Hernandez et al., 2004; Iwasaki et al., 2009).

The data presented above also support the conservation of the cap-independent mechanism of translation initiation of the insulin receptor and insulin-like growth factor receptor mRNAs in mammals. The mouse transcripts show resistance to hippuristanol under conditions that almost completely inhibit a control RNA indicating that the resistance to eIF4A inhibition is also conserved. There are no easily recognizable conserved sequence elements between the *Drosophila* and mouse UTRs, but the mode of regulation is conserved suggesting an important role for this type of translational control. This defines a functional characteristic of the insulin receptor and IGF receptor transcripts that is conserved across hundreds of millions of years of evolution (from flies to mammals).

Taken together with previous work, these data indicate that the coupling of transcription to translation of insulin receptor mRNA mediated by Foxo targets can culminate in an activated translational response. Our findings highlight a unique characteristic of the insulin receptor and IGF receptor UTRs that differentiates them from other cellular transcripts. In addition to being immune to 4E-BP, these IRESes are resistant to eIF4A inhibition. While viral IRESes are fairly common, cellular IRESes are rare and relatively unexplored. Where it has been explored, most cellular IRESes have a strong requirement for eIF4A (Thoma et al., 2004; Spriggs et al., 2009a). The INR and IGFR UTRs seem to require neither eIF4E nor eIF4A. In fact, these UTRs have the lowest identified requirement for eIF4F activity of any cellular transcript thus far. In addition, they are immune to two of the most important types of translational control, namely 4E-BP control and eIF4A inhibition. Both of these features make sense given the cellular environment when these mRNAs are to be translated. The conserved resistance to eIF4E and eIF4A inhibition of the insulin receptor transcripts should make them capable of out-competing other cellular transcripts with greater need for eIF4A or eIF4E. Using these exceptional characteristics, the insulin receptor mRNA could out-compete more abundant transcripts under times of stress or when nutrients are limiting and 4E-BP and Pdcd4 are active.

We focused on the insulin receptor UTRs as a mechanism for continued translation under conditions of general inhibition of protein synthesis as this is one of the initial components of the pathway identified as a direct Foxo target. In more recent work other components of the pathway have been identified as transcriptionally controlled by Foxo. If these targets are to be translated when Foxo is active they should also require mechanisms to escape 4E-BP and Pdcd4 inhibition. It remains to be seen if they will use the same mechanism as the INR mRNA or another mechanism.

## Materials and methods

### Fly lines

Wildtype Canton S flies are from the Bloomingtion Stock Center. *foxO^Δ94^* has been described (Slack et al., 2011).

### Analysis of Foxo genomewide data

Chromatin immunoprecipitation using Foxo antibodies followed by tiling array analysis was performed previously on starved larva (Teleman et al., 2008). The raw .CEL files for Foxo precipitated and mock precipitated arrays were downloaded from the Teleman lab web page (http://www.dkfz.de/en/signal-transduction-cancer/pages/Data.html). Triplicate samples were combined and the Foxo precipitated samples were compared to mock precipitated samples using using the Affymetrix Tiling analysis (TAS) software. Combined mock arrays were used to set the background signal intensities for the ChIP arrays. The Integrated Genome Browser (Nicol et al., 2009) was used to visualize the resultant profile.

### Cloning of mouse insulin receptor and insulin-like growth factor receptor-1 UTRs

Oligonucleotides were synthesized corresponding to annotated transcripts with the longest 5′ for both Insulin receptor (corresponding to EST G430111A11) and IGF-1 receptor (corresponding to ESTs CJ180736 and CJ173921) (Supplementary file 1). The oligos were used to clone the UTRs from cDNA derived from NIH 3T3 cells by PCR.

### Purification of his-tagged eIF4A and PDCD-4

*Drosophila* eIF4A and Pdcd4 were cloned into pET28 in frame with the 6x HIS tag from cDNA using PCR and standard cloning methods. The plasmid was transformed into BL21* (DE3) cells (Life Technologies, Grand Island, NY) containing pLacIRARE2 (Novagen, EMD Millipore, Billerica, MA). After induction with 1 mM IPTG overnight at 25°C, eIF4A or PDCD-4 was purified using HisPur Ni-NTA Resin (Thermo Fisher Scientific, Rockford, IL) according to manufacturer’s directions. PDCD-4 was eluted from the resin using 500 mM imidazole in PBS. eIF4A was eluted using 50 mM EDTA in 1X PBS. Purified 6His-Pdcd4 and 6His eIF4A was used to make rabbit polyclonal antisera (Cocalico Biologicals, Inc., Reamstown, PA).

### Pdcd4 affinity chromatography

Full length *Drosophila* Pdcd4 was cloned into pGEX2TKN in frame with GST. GST-PDCD-4 or GST alone was expressed in BL21* (DE3) cells (Invitrogen, Grand Island, NY) containing pLacIRARE2 (Novagen, EMD Millipore, Billerica, MA). Expression was induced with 1 mM IPTG overnight at 25°C. Cells were resuspended and lysed in 1X PBS with lysozyme. GST or GST-PDCD4 was immobilized on glutathione sepharose 4B (GE Healthcare Biosciences, Pittsburgh, PA). Equal amounts of recombinant eIF4A were applied to 50 µl of resin containing either GST or GST-Pdcd4. The resin was incubated for 1 hr at 4°C. The resin was poured into a small spin column (Pierce, Thermo Fisher Scientific, Rockford, IL) and washed with 100 column volumes wash buffer (20 mM Tris pH 7.5, 100 mM NaCl, 1 mM DTT, 0.1 mM EDTA, 0.1 mg/ml BSA). Bound proteins were eluted with wash buffer containing 10 mM reduced glutathione for 1 hr at 4°C. Samples were separated by SDS-PAGE, transferred to nitrocellulose and recombinant eIF4A was detected with a monoclonal antibody directed against the 6x HIS tag on eIF4A (A00186 GenScript, Piscataway, NJ) and a fluorescent secondary antibody against mouse IgG using a Li-Cor Odyssey Infrared imaging system.

### eIF4A immunoprecipitation

15 ml of a saturated culture of *Drosophila* S2 cells were harvested by centrifugation. The cells were washed once with 1X PBS. The cells were resuspended in two packed cell volumes hypotonic buffer (10 mM HEPES pH 7.4, 10 mM KCl, 5 mM MgCl_2_, 1 mM DTT, 1X protease inhibitors [Sigma-Aldrich Corp., St. Louis, MO]). Triton X-100 was added to 0.5% and the cells were left on ice 30 min. Nuclei were pelleted 10 min at 6000×*g*. Supernatant was transferred to a new tube. 10% of the sample was saved for input analysis. Polyclonal rabbit antisera to *Drosophila* eIF4A was added to the remainder of the sample and the sample was incubated at 4°C overnight with constant mixing. Samples were centrifuged 2 min at 23,000×*g*. The supernatant was combined with 50 µl protein-A Sepharose (GE healthcare) and incubated at 4°C for 2 hr. The mixture was poured into a small spin column (Pierce, #89869) connected to a needle and washed with 5 ml 1XPBS 0.1% Triton X-100 followed by 1 ml 1X PBS. Proteins were elute by addition of 2X SDS-PAGE buffer (62.5 mM Tris-HCl, pH 6.8, 25% glycerol, 2% SDS, 0.01% Bromophenol Blue, 5% β-mercaptoethanol) incubation at room temperature for 10 min and collected by centrifugation. Samples were separated by SDS-PAGE. The gel was transferred to nitrocellulose and probed with rabbit α-*Drosophila* Pdcd4 antisera directed against full length 6His-Pdcd4 (1:1000). To avoid detection of the IgG used for precipitation the immunoblot was developed with biotinylated protein-A (1:5000) (Lal et al., 2005) followed by fluorescent labeled streptavidin (1:5000) using a Li-Cor Odyssey Infrared imaging system. For experiments with the mutant Pdcd4, 10 ml of *Drosophila* S2 cells were plated at 1 × 10^6^ cells/ml in Schneider’s media supplemented with 10% FBS in a 10-cm dish. Constructs expressing wild-type myc-Pdcd4 or mutant myc-Pdcd4-282 A286A were transfected using effectene (Qiagen Inc., Valencia, CA). 4 days later the cells were harvested and immunoprecipitation of eIF4A was performed as described above except transfected Pdcd4 was detected with a monoclonal antibody directed against the myc tag (9E10) and a fluorescent secondary antibody against mouse IgG using a Li-Cor Odyssey Infrared imaging system.

### Immunoblot of Pdcd4

Protein concentrations were determined using BCA assay (Pierce) and equal amounts of protein were loaded onto a 10% SDS-PAGE gel. The gel was transferred to nitrocellulose and probed with rabbit α-*Drosophila* Pdcd4 antisera directed against full length 6His-Pdcd4 (1:500) and mouse α-tubulin antibody (1:1000).

### In vitro transcription

Transcription templates for monocistronic RNAs were created using PCR containing a template-specific forward primer with a T7 promoter incorporated and a vector specific reverse primer. Dicistronic RNA templates were made by digesting the cellular reporter vectors downstream of the firefly luciferase coding sequence and utilizing a T7 promoter incorporated in the vector. Templates were purified using column clean up protocol and eluted in 50 µl 10 mM Tris pH 8.0 (Epoch Life Science, Missouri City, TX). Templates were transcribed using T7 polymerase and subsequently purified using LiCl precipitation. Transcripts were capped using vaccinia virus capping enzyme (New England Biolabs, Ipswich, MA) as recommended and purified using RNeasy column protocol (Qiagen). Transcripts were tailed using poly(A)polymerase (New England Biolabs, Ipswich, MA) and purified using RNeasy columns (Qiagen).

### Competitive in vitro translation using *Drosophila* embryo extract

Embryo translation extracts were prepared as described from 0- to 4-hr embryos (Marr et al., 2007). Extracts were left untreated (no Micrococcal nuclease treatment) to allow translation under competitive conditions. Translation assays were performed in 6 µl of *Drosophila* embryo extract, 0.1 mM spermidine, 60 µm Amino Acids, 16.8 mM creatine phosphate, 800 ng of creatine kinase, 24 mM HEPES (pH 7.4), 0.4 mM Mg acetate, 30 mM K acetate, 1 µg of calf liver tRNA, and 100 ng of template RNA in a 10-µl reaction. Hippuristanol was added to a final concentration of 2µM or otherwise indicated. For assays containing PDCD-4, protein was added to a final concentration of 320 nM and was preincubated for 15 min with extract before the addition of RNA templates. Translation reactions were incubated at 27°C for 1 hr and luciferase activity was measured using 100 µl of luciferase substrate (Promega Corp., Madison, WI). Firefly luciferase was measured by adding 100 µl of 75 mM HEPES pH 8.0, 5 mM MgSO4, 20 mM DTT, 100 µM EDTA, 530 µM ATP, 0.5 mM coenzyme A, and 0.5 mM D-luciferin and renilla was measured by adding 100 µl 25 mM Na4PPi, 10 mM NaOAc, 15 mM EDTA, 0.5 M Na2SO4, 1.0 M NaCl, and 0.1 mM Coelenterazine, pH 5.0. All experiments were performed at least twice in triplicate.

### Competitive in vitro translation using rabbit reticulocyte extract

Translation assays were performed in 6 µl of untreated rabbit reticulocyte extract (no Micrococcal nuclease treatment to allow translation under competitive conditions). (Green Hectares, McFarland, WI), 0.1 mM spermidine, 60 µm Amino Acids, 16.8 mM creatine phosphate, 800 ng of creatine kinase, 24 mM HEPES (pH 7.4), 0.4 mM Mg acetate, 30 mM K acetate, 1 µg of calf liver tRNA, and 100 ng of template RNA in a 10-µl reaction. Translation reactions were incubated at 37°C for 30 min and luciferase activity was measured using 100 µl of luciferase substrate (Promega). For assays containing excess m7G cap, cap structure analogue (New England Biolabs, #S1407S) was added to a final concentration of 1 mM. All experiments were performed at least twice in triplicate.

### Cell culture, total RNA extraction, and RT-qPCR

*Drosophila* S2 cells with a stable transfection of constitutively active Foxo under the control of the metallothionein A promoter were maintained in Schneider’s Insect Media supplemented with 10% fetal bovine serum (Puig et al., 2003). These cells were plated at 1.25 × 10^6^ cell/ml, and expression was induced by addition of 500 µm CuSO_4_ for 16 hr. During induction the media was supplemented with 1 µg/m bovine insulin. For protein samples, cells were lysed in RIPA buffer (PBS containing 10 mM EDTA, 1% Triton X-100, 1% SDS, 1% deoxycholate, 1× complete protease inhibitor [Roche, Indianapolis, IN], 10% glycerol). Total RNA was extracted from mock-treated and induced cells using TRI Reagent according to manufacturer’s protocol (Molecular Research Center, Inc., Cincinnati, OH). 5 µg of total RNA were digested by DNaseI (New England Biolabs). First strand cDNA sythesis was done using a mix of oligo-dT and random hexamers with MMLV reverse transcriptase. The final concentrations of the cDNA reaction were 50 mM Tris-HCl (pH 8.3), 50 mM KCl, 3 mM MgCl_2_, 10 mM DTT, 400 µM dNTPs, 1–2 µg RNA, 500 ng primers, 200 units MMLV RT. cDNAs were diluted 1:10 in TE pH 8. qPCR was run using 5 µl cDNA, GoTaq qPCR Master Mix (Promega), and primers at a final concentration of 100 nM in a 20-µl reaction. qPCR was done using specific primers against *Drosophila* Pdcd4, RP49, GstD1, and 4E-BP (Supplementary file 1). Pdcd4 fold-expression was calculated as a fraction of RP49 and normalized to mock-treated expression levels. All experiments were performed at least twice in triplicate.

### Paraquat treatment

7-day-old adult male flies were starved for 5 hr and transferred to vials containing 5% sucrose or 5% sucrose+5 mM paraquat. After 24 hr flies were harvested and total RNA was extracted from mock-treated and paraquat-treated flies using TRI Reagent according to manufacturer’s protocol (Molecular Research Center, Inc.).

### Discistronic assays

Previously described dicistronic reporter constructs (Marr et al., 2007) were subcloned into a plasmid containing the metallothionein A promoter for metal inducible expression (Marr et al., 2006). The IRES sequence from HCV (Kieft et al., 1999) was subcloned into the inducible expression vector. For expression in S2 cells, Pdcd4 was cloned under a minimal actin promoter. For the Pdcd4 double mutant construct, amino acids 282, and 286 (Figure 1B) were mutated to alanine by site directed mutagenesis. S2 cells were maintained in Schneider’s media with 10% FBS and Penicillin/Streptomycin. For transfection, cells were plated at 1.25×10^6^ cells/ml in Schneider’s supplemented with 10% FBS and an additional 1 µg/ml bovine insulin. DNA was transfected at a 4:1 ratio expression plasmid to reporter plasmid using effectene transfection reagent (Qiagen) following instructions for S2 cells. Cells were induced with 500 µM CuSO_4_ 36 hr after transfection. Cells were lysed in passive lysis buffer (Promega) and assayed 36 hr after induction using a dual luciferase assay. Firefly expression was measured in 75 mM HEPES pH 8.0, 5 mM MgSO_4_, 20 mM DTT, 100 µM EDTA, 530 µM ATP, 0.5 mM coenzyme A, and 0.5 mM D-luciferin. Renilla expression was measured by addition of an equal volume of 25 mM Na4PPi, 10 mM NaOAc, 15 mM EDTA, 0.5 M Na_2_SO_4_, 1.0 M NaCl, and 0.1 mM Coelenterazine. All experiments were performed at least twice in triplicate.

### Chromatin immunoprecipitation (ChIP)

*Drosophila* S2 cells expressing constitutively active Foxo were formaldehyde crosslinked. Nuclei were isolated, lysed, and chromatin was sonicated to 500–1000 bp in length. Chromatin was incubated with polyclonal sera against full length Foxo. The chromatin/antibody mix was then incubated with protein A beads to isolate Foxo-bound chromatin from the sample. Purified DNA was assayed by qPCR to determine enrichment for genomic sites bound by Foxo. Enrichment is based on signal increase compared to a region of the genome in the first intron of CG15414 (Supplementary file 1).

### Mammalian dicistronic transfection

The mouse insulin receptor and insulin-like growth factor one receptor 5′ UTRs were subcloned into the plasmid pGLRSVRF. This plasmid contains the RSV LTR followed by the renilla luciferase open reading frame, the firefly luciferase open reading frame and the SV40 early polyadenylation signal. The UTRs were cloned between the renilla and firefly open reading frames. For transfection NIH3T3 cells were trypsinized and counted. 350 µl of cells at a concentration of 1 × 10^5^ cells per milliliter were plated in each well of a 24-well plate. Cells were transfected using Effectene (Qiagen) according to manufacturer’s instructions. Cells were lysed in passive lysis buffer (Promega) and assayed 36 hr after induction using a dual luciferase assay (Promega).

## Additional files

Supplementary file 1. Oligonucleotides used in this study. All primers written 5′ to 3′. **DOI:** http://dx.doi.org/10.7554/eLife.00542.008

### Major dataset

The following previously published dataset was used:

TelemanAA, HietakangasV, SayadianAC, CohenSM, 2008, Data from: Nutritional control of protein biosynthetic capacity by insulin via Myc in Drosophila, http://www.dkfz.de/en/signal-transduction-cancer/pages/Data.html, Publicly available at the German Cancer Research Center (http://www.dkfz.de/).
